# Simvastatin induces heme oxygenase-1 *via* NF-E2-related factor 2 (Nrf2) activation through ERK and PI3K/Akt pathway in colon cancer

**DOI:** 10.18632/oncotarget.10078

**Published:** 2016-06-15

**Authors:** Hyun Joo Jang, Eun Mi Hong, Mikang Kim, Jae Hyun Kim, Juah Jang, Se Woo Park, Hyun Wu Byun, Dong Hee Koh, Min Ho Choi, Sea Hyub Kae, Jin Lee

**Affiliations:** ^1^ Division of Gastroenterology, Department of Internal Medicine, Dongtan Sacred Heart Hospital, Hallym University School of Medicine, Gyeonggi do, Korea; ^2^ Division of Gastroenterology, Department of Internal Medicine, Hangang Sacred Heart Hospital, Hallym University School of Medicine, Seoul, Korea

**Keywords:** statin, NF-E2-related factor, colon cancer, extracellular-signal-regulated kinase, heme oxygenase-1

## Abstract

Statin has been known not only as their cholesterol-lowering action but also on their pleiotropic effects including anti-inflammatory and anti-oxidant as well as anti-cancer effect. Nrf2 (NF-E2-related factor 2) is a transcription factor to activate cellular antioxidant response to oxidative stress. There are little known whether statins affect activation of Nrf2 and Nrf2 signaling pathway in colon cancer cells. We investigated whether simvastatin stimulates the expression of Nrf2 and nuclear translocation of Nrf2 and which signal pathway is involved in the expression of Nrf2 and antioxidant enzymes. We investigated the effect of simvastatin on the expression of Nrf2 and nuclear translocation of Nrf2 in two human colon cancer cell lines, HT-29 and HCT 116 through cell proliferation assay, Western blotting and immunocytochemical analysis. We evaluated which signal pathway such as ERK or PI3K pathway affect Nrf2 activation and whether simvastatin induces antioxidant enzymes (heme oxygenase-1 (HO-1), NAD(P)H: quinine oxidoreductase 1 (NQO1), γ-glutamate-cysteine ligase catalytic subunit (GCLC)). We demonstrated simvastatin-induced dose-dependent up-regulation of Nrf2 expression and stimulated Nrf2 nuclear translocation in colon cancer cells. We also demonstrated that simvastatin-induced anti-oxidant enzymes (HO-1, NQO1, and GCLC) in HT-29 and HCT 116 cells. PI3K/Akt inhibitor (LY294002) and ERK inhibitor (PD98059) suppressed simvastatin-induced Nrf2 and HO-1 expression in both HT-29 and HCT 116 cells. This study shows that simvastatin induces the activation and nuclear translocation of Nrf2 and the expression of various anti-oxidant enzymes via ERK and PI3K/Akt pathway in colon cancer cells.

## INTRODUCTION

Colorectal cancer (CRC) is one of the most common cancers, and CRC-related deaths are highly prevalent worldwide including Korea, which has become a significant public health in most developed countries [1] Moreover, the five-year survival rate remains quite low in advanced or metastatic CRC [2]. According to the annual report of the Korea Central Cancer Registry, a total of 24,986 CRC cases detected in Korea in 2009, representing 13.0% of all incident cancer cases [3, 4]. CRC is the second and third most common cancer in Korean men and women, respectively [3, 4].

The nuclear factor-erythroid 2-related factor 2 (Nrf2) is a transcription factors which are activated in response to cellular stress. Nrf2 binds to ARE in the promoter regions of target genes for antioxidant enzymes [5–7]. Nrf2 is traditionally regarded as the cellular defense mechanism and cell survival [6–8] and has been recently expected as a novel target for the chemoprevention of CRC [8]. Many factors including oxidative stress, reactive nitrogen species (RNS) and reactive oxygen species (ROS) can cause chronic inflammation. It is currently well known that chronic inflammation is a major factor contributing to 15-20% cancers including CRC. However, recent studies have revealed the bad side of Nrf2 which promotes the survival of cancer cells as well as normal cells [9].

HMG-CoA reductase inhibitors (statins) are widely prescribed drugs used in the treatment of dyslipidemia to prevent cardiovascular or cerebrovascular events. Their therapeutic role has been known not only as their cholesterol-lowering effect but also as their pleiotropic actions which means anti-inflammatory, anti-thrombotic, vascular cytoprotection, anti-oxidant, immunomodulatory, or anti-cancer effects of statin [10, 11]. They are also known to increase atherosclerotic plaque stability, to improve endothelial function and to have an anti-oxidant effect and anti-inflammatory activity [12]. According to some studies that investigate the antioxidant and anti-inflammatory effect of statins, atorvastatin activates heme oxygenase-1 at the stress response elements in prostatic cancer cells and breast cancer cells [13]. Simvastatin decreases reactive oxygen species level by Nrf2 activation via PI3K/Akt pathway in primary mouse embryonic fibroblasts from wild-type or Nrf2 knockout C57BL6J mice and ST-2 cells [14]. Simvastatin stimulates HO-1 expression in Neuro 2A cells by Nrf2 protein [15]. Fluvastatin prevents oxidative stress in vascular smooth muscle via the Nrf2-dependent antioxidant pathway [16]. These studies have shown that statin activates HO-1 and decrease reactive oxygen species level and have anti-oxidant effect in some cancer cells, fibroblasts, and neuronal cells. In our previous study, we have also revealed that simvastatin activates the apoptosis of colon cancer cells and inhibits IGF-1 induced ERK and Akt via the downregulation of IGF-1R expression and proapoptotic ERK activation [in press]. However, there are little known how statins affect activation of Nrf2 and HO-1 antioxidant systems in colon cancer cells. In this study, we investigated the effect of simvastatin on the expression of Nrf2 and HO-1 related antioxidants in two colon cancer cell lines, HT-29, and HCT-116.

## RESULTS

### Simvastatin induces Nrf2 expression and nuclear translocation of Nrf2

**Figure 1:**
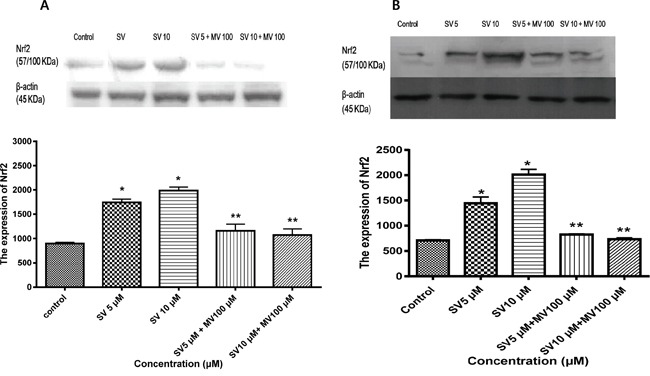
Simvastatin induces Nrf2 expression in HCT116 and HT-29 cells **A.** HCT116 cells were incubated with the indicated concentrations of simvastatin for 24 h. Nrf2 expression was determined using Western blotting. Simvastatin increases expression of Nrf2 in a dose-dependent manner (*p < 0.001 compared with control), which is reversed by mevalonate treatment (**p < 0.001 compared with simvastatin alone). SV, simvastatin; MV, mevalonic acid. **B.** HT-29 cells were incubated with the indicated concentrations of simvastatin for 24 h. Nrf2 expression was determined using Western blotting. The data represent three independent experiments. Simvastatin increases expression of Nrf2 in a dose-dependent manner (*p < 0.001 compared with control), which is reversed by mevalonate treatment (**p < 0.001 compared with simvastatin alone). SV, simvastatin; MV, mevalonic acid.

**Figure 2:**
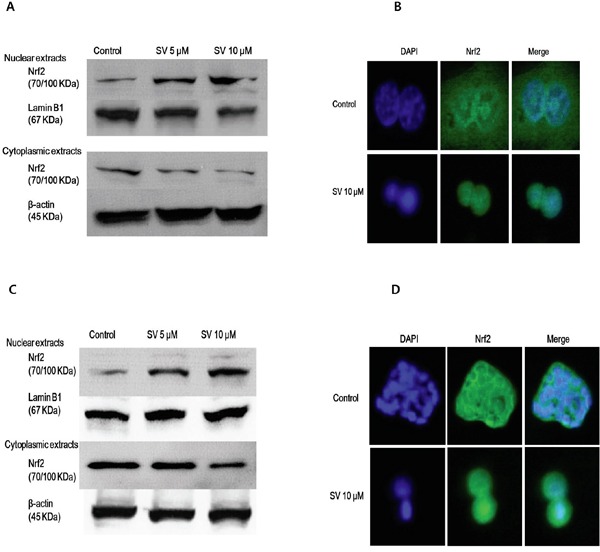
Effect of simvastatin on nuclear translocation of Nrf2 **A.** HCT116 cells were treated with 5 μM and 10 μM of simvastatin for 24 h. Nuclear fractions and cytoplasmic fractions were respectively analyzed by Western blotting for the detection of Nrf2 nuclear translocation Simvastatin increases the nuclear accumulation of Nrf2 in a dose-dependent manner. **B.** HCT116 cells were treated with 10 μM simvastatin for 24 h. After treatment, cell was fixed and labeled with an anti-Nrf2 antibody, followed by a fluorescein isothiocyanate (FITC)-conjugated secondary antibody. The nuclei were visualized by DAPI staining. Simvastatin for 24 h increased the nuclear accumulation of Nrf2 protein from the cytoplasm to the nucleus by immunocytochemical analysis. **C.** HT-29 cells were treated with the indicated concentrations of simvastatin for 24 h. Nuclear fractions and cytoplasmic fractions were respectively analyzed by Western blotting for the detection of Nrf2 nuclear translocation. Simvastatin increased the nuclear accumulation of Nrf2 in a dose-dependent manner. **D.** HT-29 cells were treated with 10 μM simvastatin for 24 h. After treatment, cells were fixed and labeled with an anti-Nrf2 antibody, followed by an FITC-conjugated secondary antibody. The nuclei were visualized by DAPI staining. Simvastatin for 24 h increased the nuclear accumulation of Nrf2 protein from the cytoplasm to the nucleus by immunocytochemical analysis. The data represent three independent experiments. SV, simvastatin.

The transcription factor Nrf2 is a member of the basic leucine zipper NF-E2 family and interacts with the ARE in the promoter of phase II detoxifying enzymes, including the gene encoding HO-1. Therefore, we examined whether simvastatin could induce Nrf2 activation. As shown in Figure 1A and 1B, treatment with simvastatin increased expression of Nrf2 in a dose-dependent manner which was reversed by mevalonate treatment in HCT116 and HT-29 cells. Next, we examined whether simvastatin could induce nuclear translocation of Nrf2 from the cytosol to the nucleus. As shown in Figure 2A and 2C, treatment with simvastatin increased nuclear accumulation of Nrf2 in a dose-dependent manner. We also examined nuclear translocation of Nrf2 using an immunocytochemistry assay in HCT116 and HT-29. Figure 2B and 2D showed that treatment of 10 μM of simvastatin for 24 h increased nuclear accumulation of Nrf2 protein from the cytoplasm to the nucleus by immunocytochemical analysis.

### Simvastatin induces Nrf2-related antioxidant expression in HCT116 and HT-29 cells

**Figure 3:**
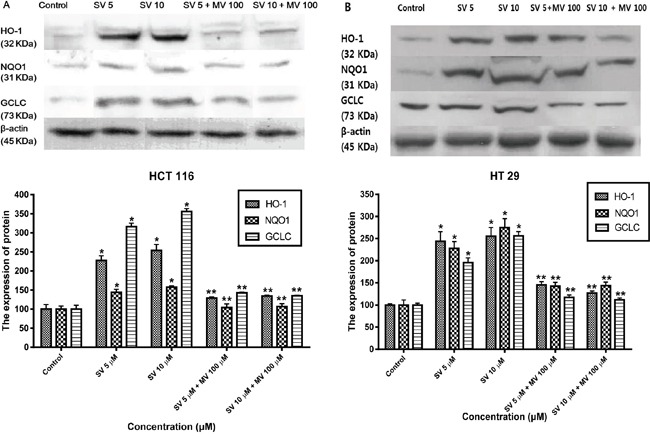
Simvastatin induces transcriptional activation of HO-1, NQO1, and GCLC **A.** HCT116 cells were treated with the indicated concentrations of simvastatin for 24 h. The expression of HO-1, NQO1, and GCLC were determined by Western blot analysis. Simvastatin increased the expression of HO-1, NQO1, and GCLC in a dose-dependent manner (*p < 0.001 compared with control), which is reversed by mevalonate treatment (**p < 0.001 compared with simvastatin alone). **B.** HT-29 cells were treated with the indicated concentrations of simvastatin for 24 h. The expression of HO-1, NQO1, and GCLC were determined by Western blot analysis. Simvastatin increased the expression of HO-1, NQO1, and GCLC in a dose-dependent manner (*p < 0.001 compared with control), which is reversed by mevalonate treatment (**p < 0.001 compared with simvastatin alone). The data represent three independent experiments. SV, simvastatin; MV, mevalonic acid.

Nrf2 binds to AREs and positively regulates the expression and induction of HO-1 and NAD(P)H: quinine oxidoreductase 1 (NQO1) genes. To investigate the effect of statins on the Nrf2–antioxidant system, we examined three representative genes: HO-1, NQO1, and γ-glutamate-cysteine ligase catalytic subunit (GCLC) by western blotting. To assess the role of simvastatin in the induction of HO-1 expression, HCT116 and HT-29 were treated for 24 h with simvastatin at doses of 5 μM and ten μM and HT-29. After treatment with simvastatin for 24 h, the expression of the HO-1 protein was increased in a concentration-dependent manner and reversed by mevalonate treatment on HCT116 and HT-29 cells (Figure 3A and 3B). We also observed that treatment with simvastatin on HCT116 and HT-29 cells led a significant increase in expression for NQO1 and GCLC with concentration-dependent manner (Figure 3A and 3B). These effects were statistically significant increases compared with the control. Thus, we speculate that the simvastatin upregulated Nrf2-related antioxidant enzymes in HCT116 and HT-29 cells.

### Simvastatin activates Nrf2 and HO-1 through ERK and PI3K/Akt pathway

**Figure 4:**
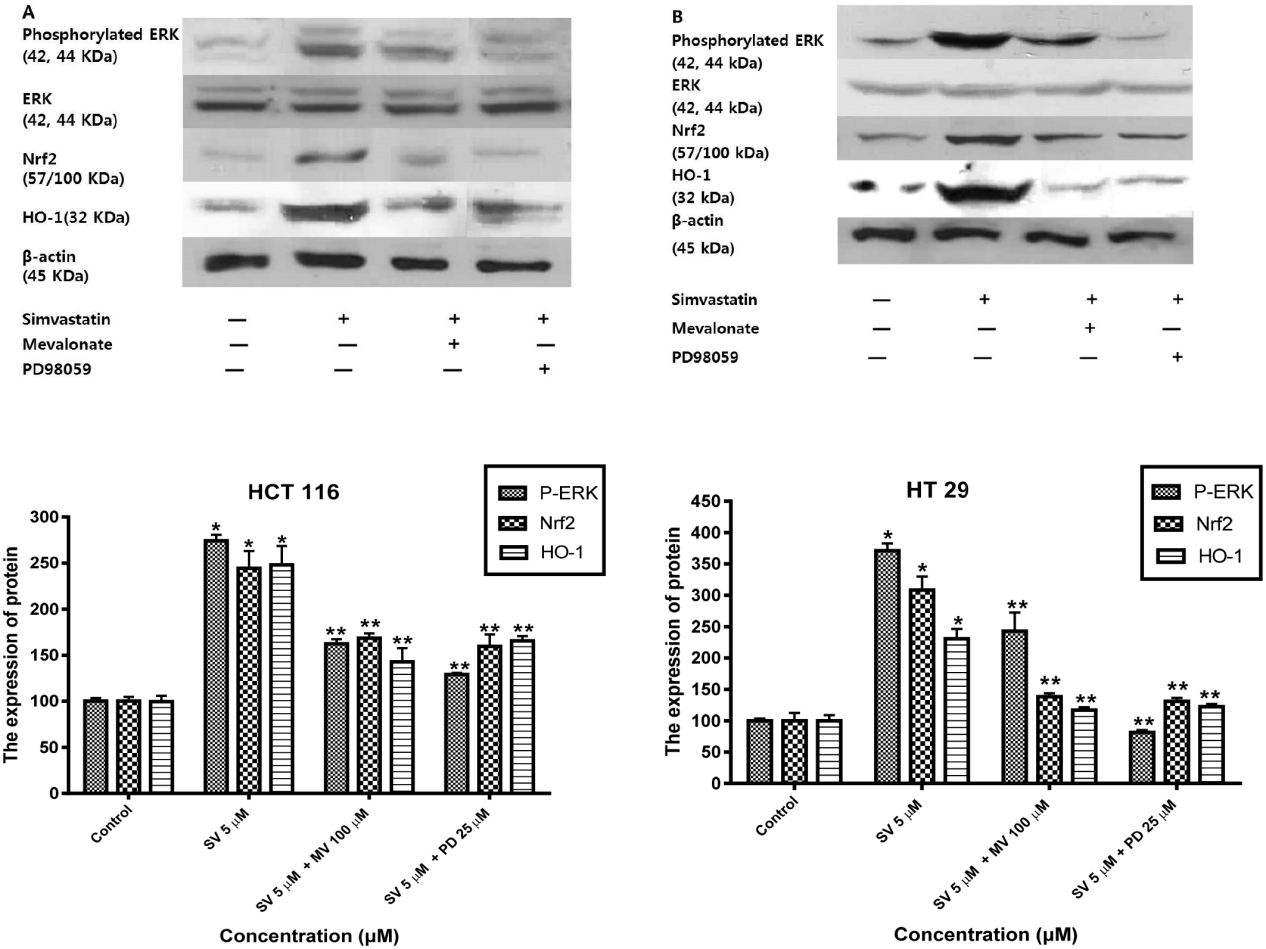
Simvastatin-induced ERK phosphorylation activates Nrf2 and HO-1 expression in HCT116 and HT-29 cells **A.** HCT116 cells were pretreated with or without PD98059 (25 μM) or mevalonate (100 μM) for 2h and then co-treated with simvastatin (5 μM) for 24 h. Western blot analysis was performed. Simvastatin increased the expression of phosphorylated ERK, Nrf2, and HO-1 in a dose-dependent manner (*p < 0.001 compared with control), which is reversed by mevalonate or PD98059 treatment (**p < 0.001 compared with simvastatin alone). SV, simvastatin; MV, mevalonic acid; PD, PD98059. **B.** HT-29 cells were pretreated with or without PD98059 (25 μM) or mevalonate (100 μM) for 2h and then co-treated with simvastatin (10 μM) for 24 h. Western blot analysis was performed. The data represent three independent experiments. Simvastatin increased the expression of phosphorylated ERK, Nrf2, and HO-1 in a dose-dependent manner (*p < 0.001 compared with control), which is reversed by mevalonate or PD98059 treatment (**p < 0.001 compared with simvastatin alone). SV, simvastatin; MV, mevalonic acid; PD, PD98059.

**Figure 5:**
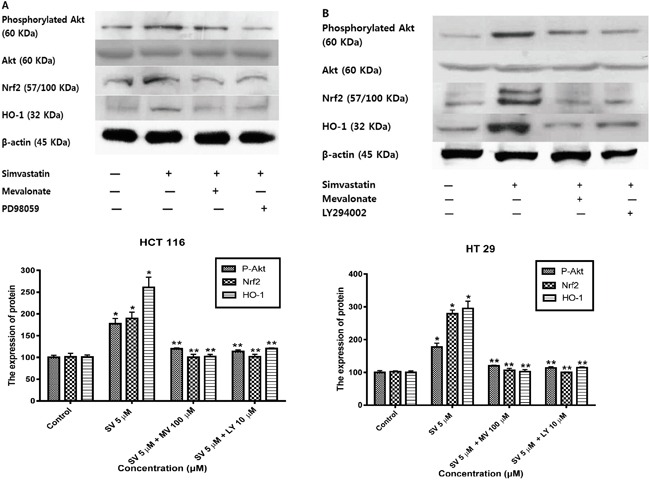
Simvastatin-induced PI3K/Akt phosphorylation activates Nrf2 and HO-1 expression in HCT116 and HT-29 cells **A.** HCT116 cells were pretreated with or without LY294002 (10 μM) or mevalonate (100 μM) for 2h and then co-treated with simvastatin (5 μM) for 24 h. Western blot analysis was performed. Simvastatin increased the expression of phosphorylated Akt, Nrf2, and HO-1 in a dose-dependent manner (*p < 0.001 compared with control), which is reversed by mevalonate or LY294002 treatment (**p < 0.001 compared with simvastatin alone). SV, simvastatin; MV, mevalonic acid; LY, LY294002. **B.** HT-29 cells were pretreated with or without LY294002 (10 μM) or mevalonate (100 μM) for 2h and then co-treated with simvastatin (10 μM) for 24 h. Western blot analysis was performed. The data represent three independent experiments. Simvastatin increased the expression of phosphorylated Akt, Nrf2, and HO-1 in a dose-dependent manner (*p < 0.001 compared with control), which is reversed by mevalonate or LY294002 treatment (**p < 0.001 compared with simvastatin alone). SV, simvastatin; MV, mevalonic acid; LY, LY294002.

Previous studies reported that mitogen-activated protein kinase (MAPK)/ERK and phosphoinositide 3-kinase (PI3K)/Akt signaling pathways are controlled by reactive oxygen species and are associated with HO-1 expression [17–20]. Therefore, we examined the possibility that the MAPK/ERK and PI3K/Akt pathways are involved in the simvastatin-induced HO-1 expression. First, we investigated whether simvastatin could activate ERK and if those signals were associated with simvastatin-induced HO-1 expression in HCT116 and HT-29. Treatment with simvastatin (5 μM in HCT-116 and ten μM in HT-29) for 24 h increased phosphorylation of ERK, an MAPK inhibitors, 25 μM PD98059 (ERK inhibitor) and mevalonate (100 μM) for two h respectively reduced simvastatin-induced Nrf2 and HO-1 expression (Figure 4A and 4B). These results indicate that ERK signaling is associated with simvastatin-induced HO-1 expression. Next, we investigated whether simvastatin increased HO-1 expression via activation of PI3K/Akt pathway. In the presence of simvastatin (5 μM in HCT-116 and ten μM in HT-29) for 24 h, Akt phosphorylation increased. Pretreatment of cells with ten μM LY294002, a PI3K inhibitor and mevalonate (100 μM) respectively inhibited simvastatin-induced Nrf2 and HO-1 expression (Figure 5A and 5B). Taken together, these results indicate that both simvastatin-induced ERK and PI3K/Akt pathways play a critical role in HO-1 induction via Nrf2 nuclear translocation in HCT116 and HT-29 cells.

## DISCUSSION

We had demonstrated that simvastatin inhibited HT-29 cell proliferation and induced apoptosis through the caspase cascade and affecting the expression of the Bcl-2 family proteins in our previous study (in press). The results of our present study demonstrated that simvastatin-induced HO-1 expression by nuclear translocation of Nrf2 via both ERK and PI3K/Akt pathway in HCT116 and HT-29 colon cancer cells.

HO-1 is a key protein that plays a central role in the cellular adaptation to stress induced by many pathological events [21]. Although the key factors participating in signal transduction mechanisms needed for transcriptional activation of HO-1 remain to be fully identified, this enzyme can be regarded as a potential therapeutic target for a variety of oxidant- and inflammatory diseases [22]. Statins induce HO-1 in several cell types, such as vascular smooth muscle cells, endothelial cells, and macrophages. The anti-inflammatory effects of statins have been linked to the induction of the cytoprotective gene encoding HO-1 [23]. Statin is known to activate the HO-1 promoter in endothelial cells and reduce free radical formation. This effect leads to their antioxidant and anti-inflammatory actions [24, 25]. The HO enzyme family degrades heme to biliverdin, thereby providing carbon monoxide (CO) and free iron (Fe2). Both bilirubin (the product of biliverdin degradation) and CO may protect molecules against oxidative stress and ischemia and reperfusion injury [26–29]. They reduce superoxide anions, suppress lipid peroxidation, prevent apoptosis, increase vasodilatation, and improve local blood flow [30, 31]. However, the role of HO-1 in cancer is controversial. HO-1 can be expressed at high levels in some tumor cells, and down-regulation of HO-1 by HO-1-shRNA or inhibition of the enzyme by specific inhibitor has been shown to inhibit proliferation of some hormone-refractory prostate cancer cells [32]. On the other hand, pro-apoptotic and anti-proliferative functions of HO-1 have been reported in prostate cancer [33], breast cancer [34] and oral cancer [35], although the mechanism of action is not known. In a previous study, HO-1 determines the differential response of breast cancer and normal cells to piperlongumine [36]. This study suggested that HO-1 has an anti-tumor function in cancer cells, but cytoprotective functions in normal cells. In our study, simvastatin activated HO-1 expression in colon cancer cells but, it is uncertain whether HO-1 expression by simvastatin protects cancer cells or inhibits cell proliferation in colon cancer cells.

Nrf2 is an important transcription factor that activates expression of antioxidant defense genes through binding to ARE in the promoter region of antioxidant genes such as HO-1 and glutathione regulatory enzymes in response to ROS [37, 38]. The activity of Nrf2 is normally suppressed in the cytosol by Keap1 protein. The treatment of cells with ARE inducers causes dissociation of Nrf2 from Keap1, allowing to Nrf2 translocate to the nucleus. In our study, we found that simvastatin-induced Nrf2 activation and nuclear translocation, which increased the expression of its downstream target enzymes, HO-1, NQO1 and GCLC in HT-29 and HCT116 cells. Nrf2 plays dual roles in cancer prevention and progression depending on the cellular context and environment. Nrf2 is considered a major mechanism of protection against chemical and radiation capable of damaging DNA integrity and initiating carcinogenesis [39]. It protects cells against chemical and radiation stress and promotes cell survival. Simvastatin activates Keap1/Nrf2 signaling in rat liver to protect the cells from the deleterious effects of oxidative stress [40]. Lovastatin protects neural stem cells against oxidative stress-induced cell death via expression of anti-apoptotic Nrf2 and PGC-1α [41]. Fluvastatin has cytoprotective effects against oxidative stress, activating antioxidant genes through Nrf2/ARE in human coronary artery smooth muscle cells [16]. In this study, simvastatin activated Nrf2 nuclear translocation, HO-1 expression, and then the expression of HO-1 related antioxidants in colon cancer cells. However, in this study, it is unclear how simvastatin-induced Nrf2 and HO-1 activation interact with apoptosis stimulated by simvastatin in colon cancer cells. It remains to investigate further study to evaluate the role of HO-1 stimulated by simvastatin in colon cancer.

Modification of the Nrf2–Keap1 complex and Nrf2 nuclear translocation is necessary to Nrf2–ARE-dependent gene expression, and several signaling pathways are associated with processes depending on cell type or stimulators [42]. The MAPKs are a family of signal-transducing enzymes participating in the regulation of many essential cellular processes. Many studies have focused on the resolution of MAPKs pathways in the activation of HO-1 in diverse cell types in response to various inducing conditions [43–46]. ERK and p38MAPK induce Nrf2 translocation and HO-1 expression through diallyl sulfide in HepG2 cells [47]. In human aortic smooth muscle cells, oxidized low-density lipoprotein induces nuclear translocation of Nrf2 via activation of MAPK [48].

A recent study showed that ERK phosphorylates Nrf2, which may induce the release of Nrf2 from the Keap1-Nrf2 complex, causing activated Nrf2 to translocate to the nucleus [49]. In other studies, PI3K/Akt has an important role in Nrf2 signaling. In response to the herb-derived phenol carnosol, Nrf2–ARE binding activity increased. A PI3K inhibitor and dominant negative mutant of Akt blocked carnosol-induced HO-1 expression in PC12 cells [48]. The present experiment designed to determine a possible role of MAPK/ERK pathway and PI3K/Akt pathway in simvastatin-induced HO-1 expression showed that simvastatin activates ERK and Akt phosphorylation. Moreover, this study demonstrated that the expression of Nrf2 and HO-1 are activated via ERK and PI3K/Akt signaling in colon cancer cells.

Taken together, we demonstrated that simvastatin activates Nrf2 activation and nuclear translocation of Nrf2 and then induces the expression of HO-1 related antioxidants via ERK and PI3K/Akt pathway in HT-29 cells. Further studies are needed to explore what the exact role of Nrf2 and HO-1 related antioxidants stimulated by simvastatin is while simvastatin suppresses cell proliferation and increases apoptosis in colon cancer cells.

## MATERIALS AND METHODS

### Materials

Dulbecco's modified Eagle medium (DMEM), fetal bovine serum (FBS), trypsin/EDTA and penicillin/streptomycin were from Gibco (Grand Island, NY, USA). Simvastatin were from Calbiochem (Gibbstown, NJ, USA), Beta-actin antibody (sc-130656, polyclonal, rabbit, 1:1000), HO-1 antibody (sc-10789, polyclonal, rabbit, 1:1000), Nrf2 antibody (sc-722, polyclonal, rabbit, 1:1000), NQO1 antibody (sc-25591, polyclonal, rabbit, 1:1000), and Goat anti-rabbit IgG-HRP (sc-2004, polyclonal, rabbit, 1:1000), were from Santa Cruz Biotechnology (Santa Cruz, CA, USA), Phopho-p44/42 MAP kinase antibody (9101s, polyclonal, rabbit, 1:1000), non-phospho-p44/42 MAP kinase antibody (9102s, polyclonal, rabbit, 1:1000), Phospho-Akt antibody (9271s, polyclonal, rabbit, 1:1000), non-phospho-Akt antibody (9272s, polyclonal, rabbit, 1:1000) were from Cell signaling (Danvers, MA, USA), GCLC antibody (ab154770, polyclonal, rabbit, 1:300) was from Abcam (Cambridge, MA, USA), and Amersham ECL™ Advance Western Blotting Detection Kit were from Amersham Biosciences (Buckinghamshire, UK).

### Cell culture

HT-29 cells were provided by Dr. C.S. Eun (Hanyang University College of Medicine, Seoul, Korea). HCT-116 cells were purchased from Korean Cell Line Bank (KCLB). Cells were cultured in DMEM with two mM glutamine and 4.5 g/L glucose supplemented with 1.5 g/L sodium bicarbonate, 10 % FBS, 100 μg/mL streptomycin, and 100 IU/ml penicillin. We exchanged the media twice a week, and the cells were kept in 37°C incubator with 5 % CO_2_. We subcultured cells when confluent (every 5~7 days) using EDTA (1 g/L) and trypsin (2.5 g/L). Experiments were carried out serum-free medium (SFM) containing 0.1 % bovine serum albumin (BSA, Sigma, St Louis, MO, USA).

### MTT (3-[4,5-dimethylthiazol-2-yl]-diphenyltetrazolium bromide) Assay

We seeded cells at a density of 5X 10^4^ cells/mL with the cultured medium in a 96-well plate. After incubation for 24 h, cells were cultured in various concentration of simvastatinin serum-free medium for 24 or 48 h. Then MTT (0.5 mg/mL) (Sigma, St. Louis, MO, USA) was added to each well and incubated for further four hour 37°C. After the medium has been removed, 100 μL of dimethylsulfoxide (DMSO) was added to each well by shaking the plate for 10 min. The optical density (OD) was evaluated by DTX 880 Multimode Detector (Beckman Coulter, Brea, CA, USA) at 570 nm. Each assay was performed in triplicates.

### Western blotting

Cells were washed with PBS and harvested with lysis buffer (50 mM Tris, pH 7.5, 150 mM NaCl, 1 mM EDTA, 1 % Tripton X-100, 1 μM phenylmethylsulfonyl fluoride (PMSF), 1 % sodium deoxycholate, 0.1 % SDS, 5 μg/ml aprotinin, 5 μg/ml leupeptin). Protein contents were analyzed using the Bradford assay (Sigma, St Louis, MO, USA). SDS-PAGE was performed with a 4 % stacking gel and a 10 % resolving gel, followed by transfer to nitrocellulose membrane (Bio-rad, Hercules, CA, USA). We blocked the membranes for one hour at room temperature in blocking solution (5 % skim milk in Tris-buffer with Tween-20 [TBS-T]: 200 mM Tris, 500 mM NaCl, pH 7.5, 0.05 % v/v Tween-20), and then incubated overnight at 4°C in 5 % BSA solution (5% bovine serum albumin in TBS-T) with Nrf2 antibody, HO-1 antibody, NQO1 antibody, GCLC antibody, beta-actin antibody, Phopho-p44/42 MAP kinase antibody, non-phospho-p44/42 MAP kinase antibody, Phospho-Akt antibody, or non-phospho-Akt antibody. The membranes were washed with TBS-T and incubated with Goat anti-rabbit IgG-HRP for one hour at room temperature. The membrane washed and incubated with Amersham ECL™ Advance Western Blotting Detection Kit for 2 min, and autoradiography was checked. The signal intensities of specific bands on the Western blotting were quantified using National Institutes of Health Image J density analysis software (Version1.43).

### Nuclear extract preparation

Nuclear extracts for western blotting and immunofluorescence staining were prepared by Nuclear/Cytosol Fractionation Kit (Biovision, Milpitas, CA, USA). Cells were washed with ice-cold PBS and collected by centrifugation at 600 x g for 5 min at 4°. We resuspended cell pellets in 0.2 mL cytosol extraction buffer A containing DTT and protease inhibitors and kept on ice for 10 minutes. The supernatant obtained was added to 11 μL of cytosol extraction buffer B, maintained on ice for 1 min, and centrifuged at 16,000 x g for 5 min. The supernatant (cytosol extract) was transferred to a new tube. The pellet (contains nuclei) was resuspended in 100 μL of nuclear extraction buffer mix, vortexed on ice for 15 seconds, and then centrifuged at 16,000 x g for 10 min. The supernatant (nuclear extract protein) concentration was measured using the Bradford method for western blotting.

### Immunofluorescence staining

Cells were cultured on glass coverslips in 6-well plates and treated with simvastatin (10 μM) for 24 h. Cells were fixed in ice cold methanol for 10 min and incubated with a Nrf2 antibody (1:100) (Santa Cruz Biotechnology, United States) in PBS containing 1% BSA for one h. After three washes with PBS, cells were incubated with the FITC-conjugated secondary antibody for one h in PBS containing 1% FBS and washed with PBS for three times. The cells on coverslips were incubated with DAPI (0.5 μg/mL) (Sigma, St. Louis, MO, USA) for 1 min. Images obtained using Olympus IX81 fluorescence microscope (Olympus, USA) and processed using Xcellence 1.2 software (Olympus, USA).

### Statistical analysis

Results from each experiment were expressed as the mean±SD of three separate experiments. Data were analyzed One-way analysis of variance (ANOVA) by Tukey's multiple comparisons tests using GraphPad Prism 4.0 software.

## Abbreviations

HO-1: heme-oxygenase-1
Nrf2: NF-E2-related factor
ERK: extracellular-signal-regulated kinase activity/protein
ARE: antioxidant responsive element
NQO1: NAD(P)H: quinine oxidoreductase 1
GCLC: γ-glutamate-cysteine ligase catalytic subunit
